# Genome-wide association study for calving performance using high-density genotypes in dairy and beef cattle

**DOI:** 10.1186/s12711-015-0126-4

**Published:** 2015-06-12

**Authors:** Deirdre C Purfield, Daniel G Bradley, Ross D Evans, Francis J Kearney, Donagh P Berry

**Affiliations:** Smurfit Institute of Genetics, University of Dublin, Trinity College, Dublin 2, Ireland; Animal & Grassland Research and Innovation Center, Teagasc, Moorepark, Fermoy, Co. Cork Ireland; Irish Cattle Breeding Federation, Bandon, Co. Cork Ireland

## Abstract

**Background:**

Calving difficulty and perinatal mortality are prevalent in modern-day cattle production systems. It is well-established that there is a genetic component to both traits, yet little is known about their underlying genomic architecture, particularly in beef breeds. Therefore, we performed a genome-wide association study using high-density genotypes to elucidate the genomic architecture of these traits and to identify regions of the bovine genome associated with them.

**Results:**

Genomic regions associated with calving difficulty (direct and maternal) and perinatal mortality were detected using two statistical approaches: (1) single-SNP (single nucleotide polymorphism) regression and (2) a Bayesian approach. Data included high-density genotypes on 770 Holstein-Friesian, 927 Charolais and 963 Limousin bulls. Several novel or previously identified genomic regions were detected but associations differed by breed. For example, two genomic associations, one each on chromosomes 18 and 2 explained 2.49 % and 3.13 % of the genetic variance in direct calving difficulty in the Holstein-Friesian and Charolais populations, respectively. Imputed Holstein-Friesian sequence data was used to refine the genomic regions responsible for significant associations. Several candidate genes on chromosome 18 were identified and four highly significant missense variants were detected within three of these genes (*SIGLEC12*, *CTU1*, and *ZNF615*). Nevertheless, only *CTU1* contained a missense variant with a putative impact on direct calving difficulty based on SIFT (0.06) and Polyphen (0.95) scores. Using imputed sequence data, we refined a genomic region on chromosome 4 associated with maternal calving difficulty in the Holstein-Friesian population and found the strongest association with an intronic variant in the *PCLO* gene. A meta-analysis was performed across the three breeds for each calving performance trait to identify common variants associated with these traits in the three breeds. Our results suggest that a portion of the genetic variation in calving performance is common to all three breeds.

**Conclusion:**

The genomic architecture of calving performance is complex and mainly influenced by many polymorphisms of small effect. We identified several associations of moderate effect size but the majority were breed-specific, indicating that breed-specific alleles exist for calving performance or that the linkage phase between genotyped allele and causal mutation varies between breeds.

**Electronic supplementary material:**

The online version of this article (doi:10.1186/s12711-015-0126-4) contains supplementary material, which is available to authorized users.

## Background

Dystocia, more commonly known as calving difficulty, is defined as a prolonged or difficult parturition, often with assistance required during delivery. The adverse effects of calving difficulty have been well documented including an increased risk of dam and calf mortality, as well as a reduction in dam and calf performance, which have a cumulative impact on herd profit [1–3]. The fact that dystocia and perinatal mortality have been included in breeding programmes demonstrates that they represent an important issue in modern-day cattle production systems [1, 4]. Globally, however, the prevalence of both calving difficulty and perinatal mortality still remains unacceptably high with values ranging from 2 to 14 % for calving difficulty and from 2 to 10 % for perinatal mortality [5–7]. Genetic variation for calving performance traits has been reported with heritability estimates ranging from less than 0.01 to 0.17 for calving difficulty and less than 0.01 to 0.12 for perinatal mortality [6, 8, 9].

Calving difficulty and perinatal mortality are complex quantitative traits, which are believed, like all traits of this nature, to be influenced by many genomic polymorphisms of individually small effect [10]. Accuracy of genomic predictions depends, in part, on the genetic architecture of the trait, which is characterized, in particular, by the number of loci that affect the trait and the distribution of the size of their effects [10]. Accurate across-breed genomic predictions rely on the presence of common genomic variants with a common substitution effect in each breed. Consequently, it is important to determine the number of variants and the size of their effects on calving difficulty and perinatal mortality to increase the accuracy of genomic predictions. Quantitative trait loci (QTL) associated with calving difficulty and perinatal mortality in cattle have been identified by genome-wide association studies (GWAS) and are mainly concentrated on chromosomes 6, 11, 12, 18 and 28 [11–13]; however, these studies focused mainly on dairy cattle breeds.

The objective of this study was to perform a GWAS based on genotypes obtained with the Illumina bovine high-density BeadChip that comprises 777 962 SNPs, to identify regions of the genome associated with three calving performance traits: (i) direct calving difficulty, (ii) maternal calving difficulty and (iii) direct perinatal mortality, in three cattle breeds (Holstein-Friesian, Charolais and Limousin) and to refine the detected genomic regions using (imputed) whole-genome sequence data.

## Methods

### Genotypic data

Illumina bovine high-density (HD) SNP genotypes were available for 2660 dairy and beef bulls, which included 770 Holstein-Friesian, 927 Charolais, and 963 Limousin animals. All animals had a genotype call rate greater than 95 %. Genotypes that were available on both the sire and son(s) were used to confirm parentage. The presence of opposing homozygous genotypes for the 1929 sire-son pairs was examined to determine Mendelian inconsistencies. Among the 777 962 high-density SNPs, 3554 autosomal SNPs with a Mendelian error rate greater than 2 % were discarded. The genotypes of sire-son pairs were set to missing for the remaining SNPs for which sporadic Mendelian inconsistencies were found. In addition, 1574 SNPs with a GenTrain score (Illumina clustering algorithm) less than 0.55, 40 934 non-autosomal SNPs and duplicate SNPs, and 17 273 SNPs with a call rate less than 95 % were also discarded. SNPs with a minor allele frequency (MAF) less than 0.02 within each breed were discarded for that breed but retained in the other breeds. Finally, SNPs that deviated (p < 0.1 × 10^−8^) from Hardy-Weinberg equilibrium within a breed were removed. After SNP editing, 605 718, 602 372 and 600 980 autosomal SNPs remained for the analysis of the Holstein-Friesian, Charolais and Limousin data, respectively. Sporadic missing genotypes were imputed using Beagle [14, 15].

### Phenotypic data

Predicted transmitting abilities (PTA) and their associated reliabilities for all 2660 dairy and beef bulls were obtained from the Irish Cattle Breeding Federation database from the December 2013 national genetic evaluation. Direct and maternal PTA for calving difficulty and direct PTA for perinatal mortality were obtained from this evaluation; there is currently no maternal component estimated for perinatal mortality in the Irish national genetic evaluations. Heritability estimates used in the national genetic evaluations were equal to 9 % for direct calving difficulty, 2 % for maternal calving difficulty, and 2 % for perinatal mortality. Irish genetic evaluations across dairy and beef breeds use multi-trait and multi-breed models. Ireland has a long history of crossbreeding including a substantial use of purebred beef sires on dairy cows (36 % of dairy cows in 2013) and also of purebred beef sires on crossbred beef cows.

In Ireland, calving difficulty is subjectively scored by producers on a linear scale of 1 to 4, where 1 = no calving assistance; 2 = slight assistance (assistance by one person, without needing to use a calf puller); 3 = considerable assistance (assistance by one person using a calf puller or more than one person); 4 = veterinary assistance (including caesarean). Perinatal mortality is recorded as a binary variable by producers, which indicates whether the calf died within a 24-h period after birth. It is a legal requirement in Ireland to record all animal deaths, including stillbirths. Phenotypic data were edited as described in detail by Purfield et al. [13]. PTA for each trait were deregressed and only sires with an adjusted reliability (i.e. reliability after removing parental contribution) of more than 30 % were retained. After editing, 2531, 1275 and 2244 animals were available for analysis of direct calving dystocia, maternal calving difficulty and perinatal mortality, respectively.

### Whole-genome association analysis

Association analyses were performed within each breed separately using two methods: single-SNP regression and a Bayesian approach.

### Single-SNP regression

Single-SNP regression (SSR) analysis was undertaken in Wombat [16] using a mixed model. Each SNP, scored as 0, 1 or 2, was included individually one at a time as a fixed effect covariate in the model. Relationships among animals were accounted for via the numerator relationship matrix. The weight on the dependent variable [17] was:

i $$ {w}_i=\frac{1-{h}^2}{\left[c+\left(1-{r}_i^2\right)/{r}_i^2\right]{h}^2}, $$

where *h*^2^ is the heritability of the trait, *r*_*i*_^2^ is the adjusted reliability of the animal and *c* is the proportion of genetic variation that is not explained by SNPs and set at 0.9 for the SSR analyses. After testing various values of *c* (i.e. 0.1, 0.2, 0.8 and 0.9) that all had a minimal impact on the results, a value of 0.9 was chosen because it allowed defining a weight for each SNP in order to attribute up to 10 % of the genetic variance to calving performance traits. Boddihireddy et al. [18] also documented no difference in prediction accuracy when comparing *c* values of 0.1, 0.5 and 0.7. Test statistics for all SNPs were obtained. Multiple-testing correction was applied using the Bonferroni correction method and p-values were transformed into their corresponding q-values assuming a false discovery rate of 5 % [19].

Within-breed SSR p-values for each SNP were combined, within trait, using the weighted Z-score method. The combined weighted Z-score was calculated as:

ii $$ Z=\frac{{\displaystyle {\sum}_i{Z}_i{w}_i}}{\sqrt{{\displaystyle {\sum}_i{w}_i^2}}}, $$

where the weight *w*_*i*_ was the square root of the sample size of breed *i* and ${Z}_i={\varPhi}^{-1}\left(1-\frac{p_i}{2}\right)$ * (*±*1 for the sign of the direction of the effect for each breed *i*) where Φ is the standard normal cumulative distribution function and *p*_*i*_ is the p-value for that SNP in breed *i*. All SNP *Z*-statistics per breed (*Z*_*i*_) were corrected for multiple-testing prior to combining the values together. This multiple-testing correction involved dividing each *Z*-statistic per breed by a lambda factor, computed as the median of all *Z*-statistics in breed *i* divided by the expected median of the *Z*-statistics under the null hypothesis of no association in breed *i*. Weighted *Z*-scores were then transformed into their corresponding p-values.

### Bayesian analyses

The Bayesian approach was carried out by fitting all SNPs simultaneously in a two-step Bayes procedure based on two different Bayesian models: (1) BayesC [20] followed by (2) BayesB [21]. First, BayesC was implemented to provide an accurate estimate of the genetic and residual variance for each trait to be included in the BayesB model since BayesB is sensitive to the prior distribution of the genetic variance and an inaccurate estimate could impact the results. BayesB assumes that many SNPs will have no association with the phenotype. Therefore, BayesB depends on the prior probability of a SNP having no association with the phenotype under investigation (π) and will fit a mixture distribution that assumes that each SNP comes from a continuous distribution or a distribution towards zero [21]. The π value was estimated as 1 minus half the number of animals in the population divided by the total number of SNPs included in the analysis.

BayesB used the posterior genetic and residual variance estimates from BayesC and the calculated π value. The chain length for all Bayesian analyses was 50 000 iterations, with the first 10 000 iterations discarded as burn-in. The dependent variable was weighted as described previously but the value of *c* (proportion of genetic variation that is not explained by SNPs) was assumed to be 0.1. Since a Bayesian method simultaneously fits all SNPs into the model, a value of 0.1 was chosen so that the genotypes account for 90 % of the genetic variation. All Bayesian algorithms were applied using GenSel, a web-based program (https://pods.iplantcollaborative.org/wiki/display/DEapps/GenSel) developed by Fernando and Garrick [22] within each breed separately.

Bayes factors (BF) were used to quantify the strength of the posterior QTL probabilities for all loci [23] using the formula:

iii $$ \mathrm{B}\mathrm{F}={\scriptscriptstyle \raisebox{1ex}{$\frac{ \Pr \left({H}_1\Big|y\right)}{1- \Pr \left({H}_1\Big|y\right)}$}\!\left/ \!\raisebox{-1ex}{$\frac{Pr\left({H}_1\right)}{1- Pr\left({H}_1\right)}$}\right.}. $$

*Pr* (*H*_*1*_) was calculated as 1-π (i.e., the probability of a SNP having no association with the phenotype) and the proportion of post-burn-in iterations that included the SNP in the model was used as evidence for an association. Bayes factors indicate the strength of an association based on a range of values: a BF greater than 3.1 indicates ‘substantial evidence’ that the SNP is associated with a QTL, a BF greater than 10.1 indicates ‘strong evidence’ and greater than 30.1 ‘very strong evidence’ [24]. The proportion of genetic variance that each SNP contributed was calculated by dividing the genetic variance attributed to that SNP by the posterior mean estimate of the genetic variance of the trait.

### Bioinformatics

Haplotypes were identified within a 100-kb region up and downstream of each SNP for which a BF greater than 200 was detected in the three breeds for either direct calving difficulty or maternal calving difficulty, or greater than 60 for perinatal mortality. These thresholds were chosen to restrict the haplotype analysis to the strongest associations identified for each trait. Gene search using Ensembl (http://ensembl.org) and NCBI map viewer (http://www.ncbi.nlm.nih.gov/mapview/) on the UMD 3.1 genome build was then completed by focusing on linkage-disequilibrium (LD) blocks that contained the SNPs of interest. QTL regions were compared to previously reported QTL in CattleQTLdb (http://www.animalgenome.org/cgi-bin/QTLdb/index) for calving difficulty or perinatal mortality as well as potentially correlated traits such as birth weight and animal size.

### Sequence analysis

Whole-genome sequence data from the 1000 bull genomes project [25] (http://www.1000bullgenomes.com/) (run 4) were available for 311 Holstein-Friesian bulls, of which 64 also had high-density genotypes. The 1000 bull genomes project identified 35.2 million SNPs across the bovine genome. The average genome coverage was 11.0X. Sequence data for the Holstein-Friesian animals used in our GWAS analysis were imputed for significant regions of the genome that were identified to be associated with calving performance in the Holstein-Friesian population analysed here. Significant regions were chosen based on clear peaks of association identified in the high-density GWAS with –log_10_ p-values greater than 8 (chromosomes 18, 2 and 4) or else based on results from previous studies which suggested the presence of QTL that affect calving performance on the chromosomes with peaks (chromosomes 6 and 10) [12, 26]. Therefore, where possible (i.e., for regions that were not located at the end of chromosomes), a 10-Mb region flanking the strongest peaks of association on each of these chromosomes (chromosome 2: 6.6 Mb, chromosome 4: 37.85 Mb, chromosome 6: 72.02 Mb, chromosome 10: 101.72 Mb and chromosome 18: 57.58 Mb) was imputed to sequence depth and re-analysed using SSR in WOMBAT as previously described. A concordance of more than 99.6 % was found between high-density genotypes and sequence data on the 64 animals for which both sources of information were available. Post-Beagle imputation accuracy was on average greater than 95 % and all SNPs were retained for analysis. Due to the limited number of Charolais and Limousin bulls for which whole-genome sequence data were available, imputation to sequence depth was deemed to be insufficiently accurate since a very large proportion of SNPs were found to be monomorphic within these breeds (~10 % of SNPs within an imputed region were polymorphic with an average r^2^ value of 0.17).

Any potential missense variants that were identified within genomic regions of strong association were analysed by SIFT (sorting intolerant from tolerant) [27] and PolyPhen (polymorphism phenotyping) [28] to predict their pathogenicity. The variant effect predictor from Ensembl (http://www.ensembl.org/info/docs/tools/vep/index.html) was used to provide SIFT scores where possible and PolyPhen scores were computed in our study.

## Results

### Direct calving difficulty

Direct calving difficulty refers to the characteristics of the calf itself (e.g., body size) and its impact on the birth process. Several SNP associations for direct calving difficulty were detected for each of the three breeds, although the exact location and direction of the effects of these associations differed between breeds. Irrespective of statistical significance, estimated allele effects had opposite signs at 283 042 SNPs for the Holstein-Friesian and Charolais populations, at 285 133 SNPs for Holstein-Friesian and Limousin populations and at 284 509 SNPs for the Charolais and Limousin populations.

**Fig. 1:**
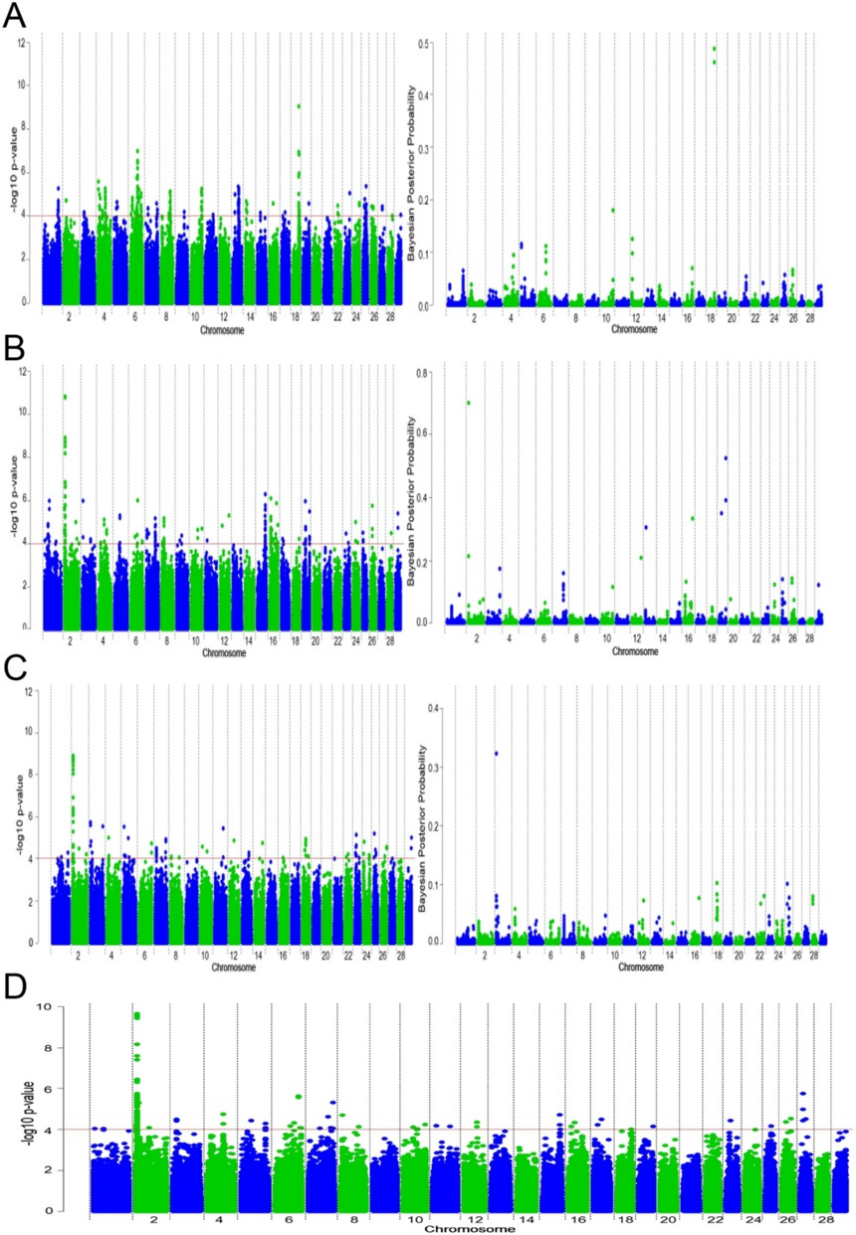
Manhattan plots for single-SNP regression and Bayesian analyses for **(a)** Holstein-Friesian, **(b)** Charolais, **(c)** Limousin and **(d)** the meta-analysis for direct calving difficulty

Twenty-three SNPs located on chromosomes 18 (10 SNPs) and 6 (13 SNPs) had a q-value less than 0.05 for direct calving difficulty in the Holstein-Friesian population. SNP ARS-BFGL-NGS-109285, located on chromosome 18, exhibited the strongest association with direct calving difficulty (Fig. 1a) in both the SSR and Bayesian analyses (Bonferroni corrected p = 5.5 × 10^−4^; q = 2.0 × 10^−4^; BF = 1684.26). SNP ARS-BFGL-NGS-109285 and an adjacent SNP, BovineHD180001676, that was 41 kb away, were cumulatively included in the Bayesian model in 94.96 % of the Gibbs chains and together they accounted for 2.49 % of the genetic variance in direct calving difficulty. Alleles of both SNPs were in complete LD. Only 0.84 % of the Holstein-Friesian animals were homozygous AA for SNP ARS-BFGL-NGS-109285 and these animals had, on average, a greater PTA for direct calving difficulty (6.42 units; s.d. 4.43) than heterozygous (4.10 units; s.d. 1.22) and homozygous animals CC (3.42 units; s.d. 0.42).

**Fig. 2:**
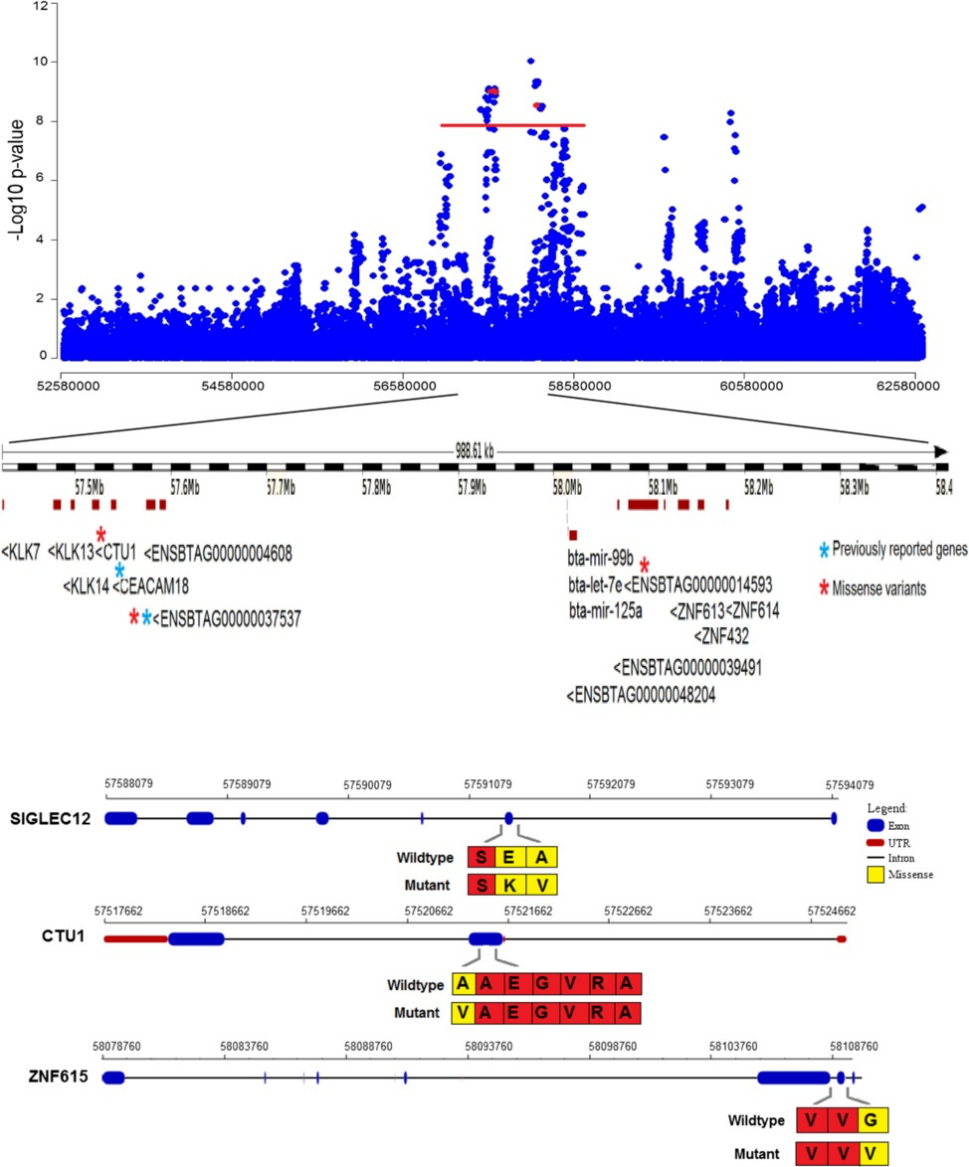
Single-SNP regression results using imputed whole-genome sequence data for a 10 Mb region around SNP ARS-BFGL-NGS-109285 on chromosome 18. Genes annotated (*) were those identified within the top 100 significant SNPs of which four were missense variants. Two of these missense variants (indicated in red in the top plot) were located in the *SIGLEC12* (ENSBTAG00000037537) gene and one each in the *CTU1* and *ZNF615* (ENSBTAG00000014593) genes

Due to the moderate proportion of genetic variation in direct calving difficulty accounted for by the genomic region between 52.58 and 62.58 Mb on chromosome 18, this region was further investigated using imputed sequence data and found to include 85 869 SNPs as illustrated in Fig. 2. SSR of the imputed sequence data revealed several peaks of association among which the peak corresponding to the interval between 57.4 and 58.4 Mb exhibited the strongest SNP associations (p < 2.5 × 10^−8^). The SNP that was most strongly associated with direct calving difficulty (p = 9.3 × 10^−11^) was located ~150 bp away from three micro-RNAs, i.e. bta-mir-99b, bta-let-7e and bta-mir-125a. Sixteen genes and gene products contained SNPs with a p-value less than 2.5 × 10^−8^ and four of these were classified as missense variants, which were distributed across three genes; two were detected in the *SIGLEC12* mRNA, one in the *CTU1* and one in the *ZNF615* gene (Fig. 2). The missense variant in *CTU1* exhibited the strongest association (p = 8.9 × 10^−10^). All four variants were classified as ‘tolerated’ based on SIFT scores, although the missense variant in *CTU1* was at the limit of this category (SIFT score = 0.06). PolyPhen scores indicated that the amino acid substitution alanine to valine in the *CTU1* missense variant could be deleterious (PolyPhen score = 0.95). The remaining detected missense variants were classified as ‘benign’ substitutions by PolyPhen.

Analysis of whole-genome sequence data for genomic intervals that flanked significant SNPs on chromosomes 6 (67.02 Mb to 77.02 Mb) and 10 (96.72 Mb to 104.30 Mb), for direct calving difficulty was also undertaken [See Additional file 1: Table S1]. Among the 61 187 SNPs on chromosome 6, 548 had a p-value less than 0.0001 with a significant peak detected at 72.02 Mb (p-value = 9.91 × 10^−8^) [See Additional file 2: Figure S1]; all but 25 of these 548 SNPs were intergenic variants. No significant missense variants were detected within this region, although a non-coding exonic variant in the novel gene *ENSBTAG00000004082* was strongly associated with direct calving difficulty (p = 7.02 × 10^−6^). Other possible candidate genes detected included the *CORIN* gene which encodes a serine protease, and the *CLOCK* gene which functions as a maternal mRNA that regulates events in the oocyte and pre-implantation embryo. Similarly, no significant missense variants were detected for chromosome 10 [See Additional file 2: Figure S1] and only 145 out of 68 650 SNPs had a p-value less than 0.0001, all of which were intergenic variants.

Both the SSR and Bayesian methods detected SNP associations on chromosome 2 for direct calving difficulty in the Charolais population (Fig. 1b and [see Additional File 1: Table S1]). The strongest association for direct calving difficulty detected with both methods was located in a region around position 5.73 Mb (Bonferroni corrected p = 9.57 × 10^−6^; q = 4.65 × 10^−6^; BF = 3310.78). Eight SNPs within this region between 5.51 and 5.73 Mb on chromosome 2 remained significant after Bonferroni correction (p = 9.97 × 10^−6^ to 0.004). The SNP with the strongest Bayesian association was included in the model for 70.17 % of the Gibbs chains and accounted for 3.13 % of the genetic variation in direct calving difficulty in the Charolais breed. Only 0.9 % of the Charolais animals were homozygous AA for this SNP and a greater PTA for mean calving difficulty was observed for these animals (9.5 units; s.d. 1.85) than for the heterozygous (8.55 units; s.d. 0.19) and homozygous animals GG (7.26 units; s.d. 0.09). This SNP was located within an intron of the *TMEM194B* gene surrounded by three other possible candidate genes (*MFSD6*, *INPP1* and *HIBCH*). In the Limousin breed, a strong association with direct calving difficulty was also detected on chromosome 2 (Fig. 1c) at a location similar to the region detected for the Charolais breed in the SSR analysis. Thirty-eight SNPs in the region between 5.89 and 6.68 Mb on chromosome 2 remained significant after correction for multiple-testing; the Bonferroni p-values of these SNPs ranged from 7.69 × 10^−4^ to 0.05 (q = 2.67 × 10^−5^ to 3.41 × 10^−3^). Several *growth factor* genes and the *myostatin* gene reside within this genomic region. In the Limousin breed, additional Bayesian associations were also identified on chromosome 3 [See Additional file 1: Table S1] within an intron of the *DCAF6* gene. Two strong Bayesian associations were also detected on chromosome 19 in the Charolais population [See Additional file 1: Table S1], located at ~120 kb from the *PRKCA* gene and accounted for 1.42 % of the genetic variation in direct calving difficulty.

### Meta-analyses for direct calving difficulty

The strongest association for direct calving difficulty detected by the meta-analysis across the three breeds was a 1-Mb region between 5.6 and 6.6 Mb on chromosome 2 (adjusted-p < 1.78 × 10^−6^) (Fig. 1d). Eleven genes (*TMEM194B*, *INPP1*, *MFDS6*, *C2H2orf88*, *HIBCH*, *MSTN*, *PMS1*, *ORMDL1*, *OSGEPL1*, *ANKAR* and *ASNSD1*) [See Additional file 1: Table S2] are present in this 1-Mb region. Single-SNP regression of imputed Holstein-Friesian sequence data for this region revealed no obvious association with direct calving difficulty [See Additional file: 2 Figure S1], which suggests that this association on chromosome 2 was mainly found for beef breeds and thus, that the meta-analysis may be influenced by the strong associations detected within these breeds.

### Maternal calving difficulty

**Fig. 3:**
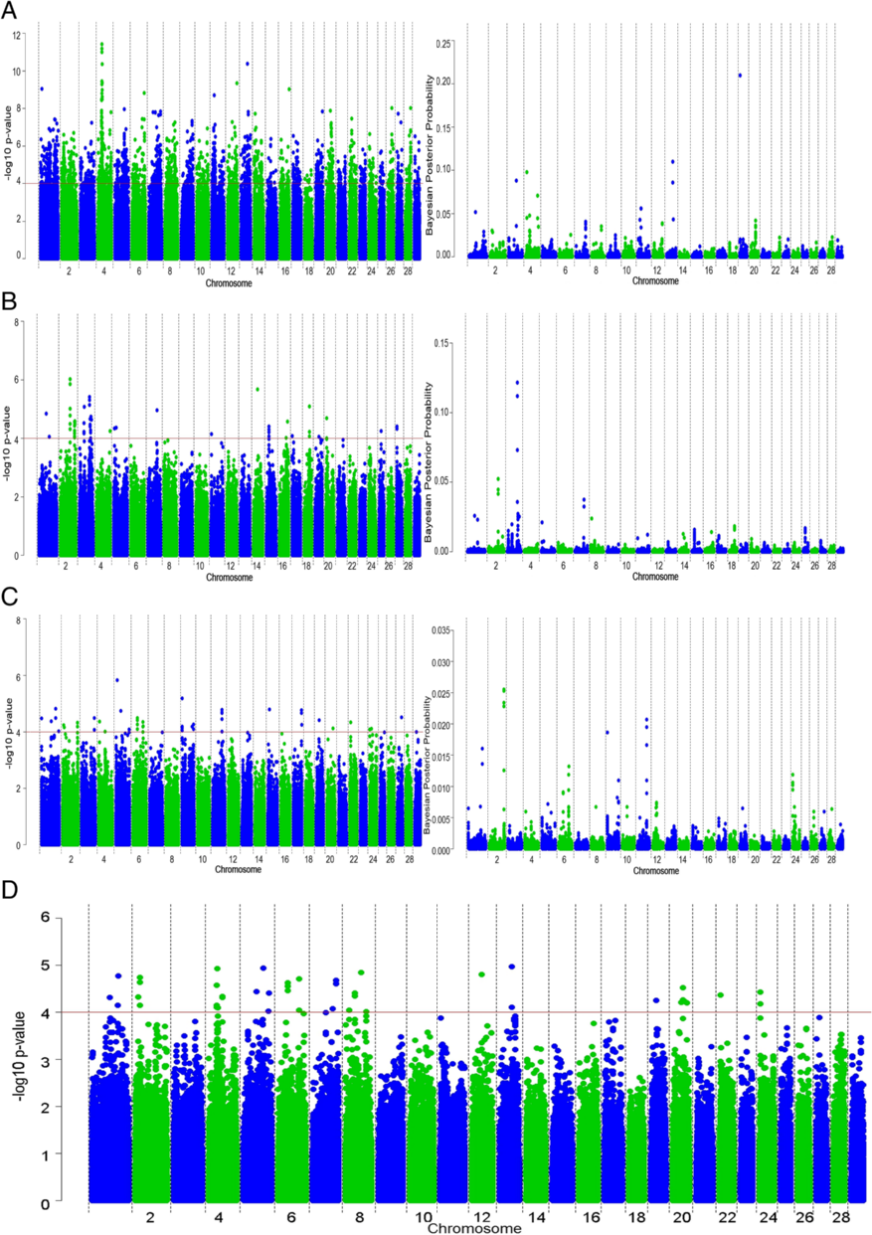
Manhattan plots for single-SNP regression and Bayesian analyses for **(a)** Holstein-Friesian, **(b)** Charolais, **(c)** Limousin and **(d)** the meta-analysis result for maternal calving difficulty

Maternal calving difficulty describes the characteristics of the dam giving birth (e.g., pelvic dimensions) and its impact on the parturition process. We found that maternal calving difficulty is influenced by many polymorphisms, each of small effect, since the maximum proportion of genetic variation accounted for by any one SNP was 0.13 % (BovineHD1900018551 on chromosome 19 for the Holstein-Friesian population). Several genomic regions associated with maternal calving difficulty in the Holstein-Friesian population were primarily identified on chromosomes 1, 4, 11, 13, and 19 (Fig. 3a). The same genomic region on chromosome 13 (around 56.55 Mb) that was significantly associated with maternal calving difficulty was detected with both association approaches (Bonferroni corrected p = 2.53 × 10^−5^; q = 2.53 × 10^−6^; BF = 220.36); however, no candidate gene was identified in this region. The four strongest associations in the SSR analyses were detected within a 20-kb region around position 37.85 Mb on chromosome 4 (Bonferroni corrected p = 2.25 × 10^−6^ to 5.99 × 10^−6^). Using imputed sequence data within the interval between 32.85 and 42.85 Mb, the same region with the strongest association was identified around 37.85 Mb [See Additional file 2: Figure S1]. The strongest SNP association (p = 3.71 × 10^−12^) was located in an intron of the *PCLO* gene. Additional candidate genes were identified within this 10-Mb region that contained intronic SNPs with p-values less than 2.51 × 10^−7^, among which were the *SEMA3D*, *HGF*, a novel gene *ENSBTAG00000019276*, *CROT* and *KIAA1324-like* genes. Two missense variants located in the *PCLO* gene and one in the *KIAA1324-like* gene had a p-value less than 0.005. SIFT scores were not available for the missense variants in the *PCLO* gene but the variant within the *KIAA1324-like* gene was classified as tolerated (SIFT = 0.38).

SSR and the Bayesian analyses identified associations with maternal calving difficulty in the Charolais breed on chromosomes 2, 3, and 7 (Fig. 3b and [See Additional file 1: Table S1]). However, after adjustment for multiple-testing in the SSR analysis, no association remained significant. Similarly, in the Limousin breed, no associations remained significant after multiple-testing adjustment, although strong SSR associations were detected on chromosome 5. In the Charolais dataset, three SNPs were detected on chromosome 3 in the region around 90.59 Mb with a BF greater than 200 and they were cumulatively included in the Bayesian model in 36.43 % of the iterations, accounting for 0.42 % of the genetic variation in maternal calving difficulty. No candidate genes near these SNPs on chromosome 3 were identified.

### Meta-analysis of maternal calving difficulty

The strongest association detected by the meta-analysis of maternal calving difficulty was on chromosome 13 (adjusted p = 1.09 × 10^−5^) (Fig. 3d) close (<2kb) to the *SIRPA* gene. The *PDYN* gene that encodes a hormone involved in signal transduction and cell communication was located 30 kb upstream.

### Perinatal mortality

**Fig. 4:**
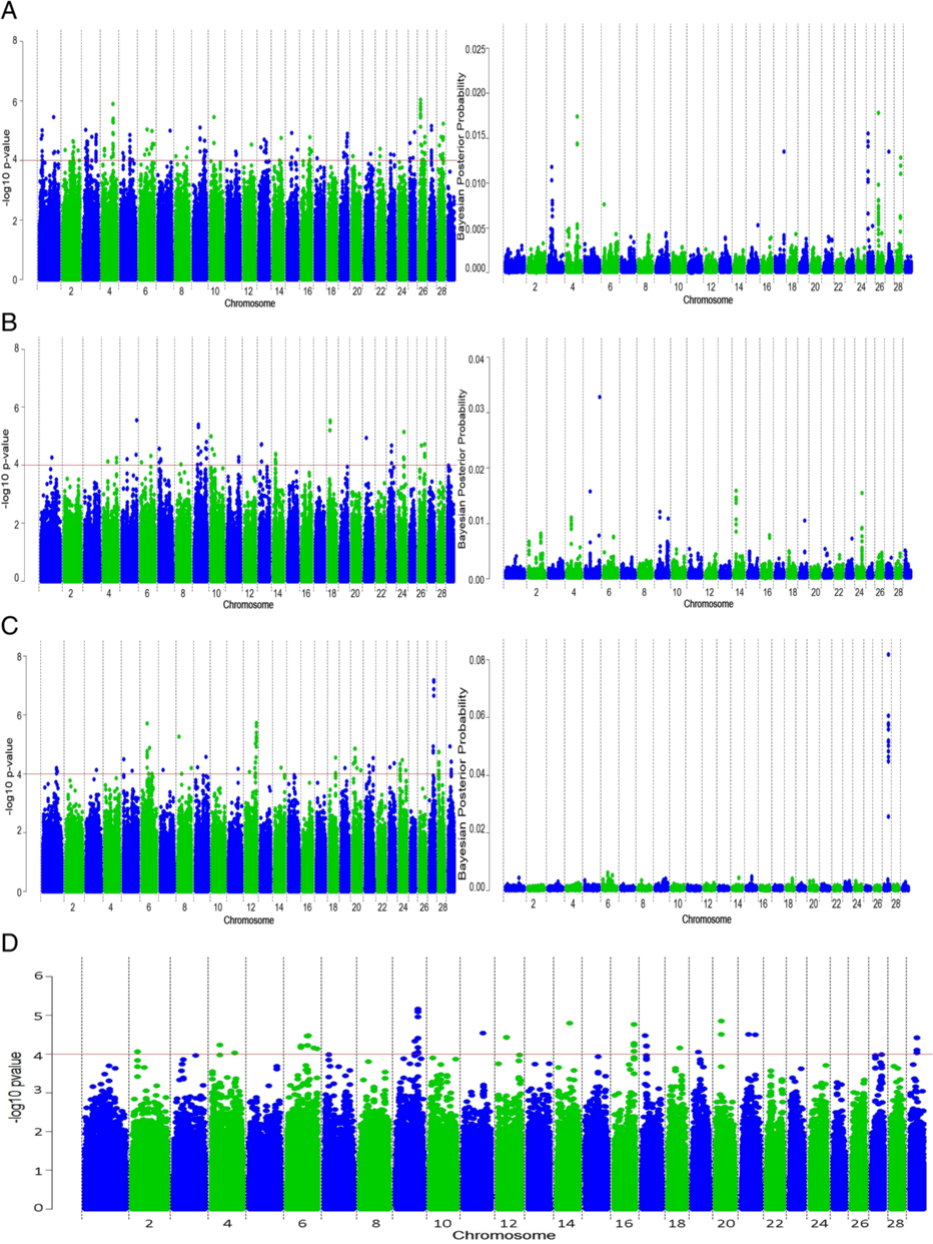
Manhattan plots for single-SNP regression and Bayesian analyses for **(a)** Holstein-Friesian, **(b)** Charolais, **(c)** Limousin and **(d)** the meta-analysis for direct perinatal mortality

Perinatal mortality in cattle is defined as calf mortality shortly before, during, or after parturition within a 24-h period. Since no maternal genetic effects are estimated in the national genetic evaluations, our analyses are based on PTA for direct mortality. Both SSR and Bayesian analyses detected the strongest associations on chromosomes 4 and 26 for the Holstein-Friesian breed ([See Additional file 1: Table S1] and Fig. 4a). Nine SNPs had a BF greater than 30.1, which provides strong evidence for an association; combining these SNPs together accounted for 0.13 % of the genetic variation in perinatal mortality in the Holstein-Friesian population. We did not perform an analysis with imputed sequence data since no association remained significant after adjustment for multiple-testing in the Holstein-Friesian dataset. For the Charolais breed, the strongest SSR and Bayesian association was on chromosome 5 (unadjusted p = 2.83 × 10^−6^; BF = 52.13). SSR analysis also detected associations on chromosomes 9 and 18 but after adjustment for multiple-testing, none remained significant. Six candidate genes (*HDAC10*, *MAPK12*, *MAPK11*, *PLXNB2*, *DENND6B*, and *PPP6R2*) were identified in this region on chromosome 5.

For the Limousin breed, both SSR and Bayesian analyses detected several strong SNPs associations with perinatal mortality on chromosome 27. Eleven SNPs had a BF greater than 60 [See Additional file 1: Table S1] and were all located within the same LD block with D' values greater than 0.91 for each pairwise combination of SNPs. These 11 SNPs were cumulatively included in the model for 63.25 % of the Gibbs chains and accounted for 0.60 % of the genetic variation in perinatal mortality in the Limousin population. The same SNPs were also highly significant in the SSR analysis and had a q-value of significance less than 0.05; three of these SNPs (BovineHD4100018377, BovineHD2700010577 and BovineHD2700010580) remained significant (p < 0.05) after Bonferroni correction. Gene annotation revealed that the *SLC20A1* gene, which encodes a phosphate transporter, was located within the LD block of these 11 SNPs.

### Meta-analysis of perinatal mortality

The strongest association with perinatal mortality across all breeds was detected on chromosome 9 (adjusted p = 6.91 × 10^−6^; Fig. 4d) and was located within the *UST* gene.

## Discussion

This study identified a large number of SNPs that were strongly associated with calving performance traits in dairy and beef cattle and across multiple breeds. Most of these regions differed between breeds, which indicates the existence of breed effects for calving performance. Many associations of small effect size were also detected for all traits, which suggests that the infinitesimal model is valid for calving performance. Nevertheless, strong genomic associations on chromosomes 18 and 2 were detected for direct calving difficulty, although a low MAF was found for the allele associated with greater calving difficulty, which suggests that prior selection occurred against calving difficulty.

### Direct calving difficulty

Our finding that SNP ARS-BFGL-NGS-109285 located on chromosome 18 was the most strongly associated SNP with direct calving difficulty in the Holstein-Friesian population in both the SSR and Bayesian analyses confirms previous analyses on two separate dairy populations genotyped at a lower density [11, 29] and on another Irish population [13] from which 628 animals were included in our study. This SNP and the adjacent SNP, BovineHD18001676, accounted for a sizeable portion (2.49 %) of the genetic variation in direct calving difficulty in the Holstein-Friesian population, which suggested that an underlying causal mutation was located within this region. Several candidate genes were identified, of which two were previously proposed to be associated with direct calving difficulty i.e. the *SIGLEC12* (also known as *SIGLEC5*) gene [11] and the *CEACAM18* gene [30]. The detection of highly significant missense variants in three of the genes (*SIGLEC12*, *CTU1* and *ZNF615*) within this region could lead to the identification of causal mutations. Their impact on protein function was predicted and, although results should be interpreted with caution because of the low specificity of Polyphen and SIFT [31], the mutation in the *CTU1* gene is the most likely candidate for direct calving difficulty in the Holstein-Friesian population. The relevance of *CTU1* in calving performance is unknown but it has been suggested that deletion of *CTU1* may affect proteins with a distinctive codon usage enriched for the AAA, GAA and CAA codons [32]. Thus, alteration of this *CTU1* gene may have an impact on regulatory proteins or genes associated with direct calving difficulty. Furthermore, the association of intergenic variants on chromosomes 6 and 10 with direct calving difficulty suggests that unidentified gene products such as non-coding RNAs, promoters, or enhancers may affect calving performance. Further work is needed to determine the exact causal mutations for direct calving difficulty in the Holstein-Friesian population.

The strong association for direct calving difficulty in both Charolais and Limousin populations that was detected on chromosome 2 (between 5.6 and 6.6 Mb) suggests that this region contains a QTL for this trait in beef breeds. Eleven candidate genes were identified within this 1-Mb region including the *myostatin* gene, which contributes to muscle hypertrophy. It has been clear for a long time that myostatin is associated with calving difficulty and homozygous animals for the double muscle mutation have a 19 % greater risk of calving difficulty [33] than heterozygous animals. In addition, selective sweeps have been reported for this QTL in the Blonde d’Aquitaine beef breed which may reflect the strong recent selection of this breed for growth and development [34]. However, our analysis of imputed Holstein-Friesian sequence data indicates no association of this QTL with direct calving difficulty in this breed. This means that the association of this genomic region with direct calving difficulty is specific to the beef breeds, for which selection has been focused mainly on growth and development.

Overall, the identification of polymorphisms that accounted for a large proportion of the genomic variation in direct calving difficulty across all breeds suggests that genomic selection algorithms should facilitate the modelling of alleles with small as well as large effects. It has been reported that Bayesian selection methods may be more suited to model the underlying genomic architecture of direct calving difficulty since they facilitate the modelling of heterogeneity in SNP contributions to the genetic variance [21].

### Maternal calving difficulty

In spite of the small size of the population analysed for maternal calving difficulty, our results suggest that this trait may be influenced by many polymorphisms each of small effect since the maximum proportion of the genetic variation accounted for by any one SNP was 0.13 %. Accuracy of genetic merit prediction has been shown to be greater for traits that are affected by SNPs with large effects, than for traits that are affected by many SNPs with small effects [10]. Thus, achieving a high level accuracy of genomic predictions for maternal calving difficulty may be more difficult due to its underlying genomic architecture.

A clear association between a region on chromosome 4 and maternal calving difficulty in the Holstein-Friesian population was detected, but for the beef breeds, we found no significant regions after adjustment for multiple-testing. This may be due to the smaller number of animals analysed for this trait (221 Charolais and 357 Limousin). However, the unadjusted significant SSR associations for the Charolais and Limousin breeds were positioned near or located at the same positions as previously reported QTL associated with maternal calving ease, birth weight, or calf size on chromosomes 3 and 2, respectively [13, 29, 35, 36]. This suggests that genomic regions involved in maternal calving difficulty may still be identified for both these breeds.

The strongest SSR association that was detected using both Holstein-Friesian high-density genotype data and imputed sequence data on chromosome 4, was located 4.3 Mb away from a QTL, which was previously associated with maternal calving difficulty in Danish and Swedish Holstein cattle [29]. Although none of the missense variants detected in this region were predicted to have an impact on protein structure, strong associations with intronic variants of the *PCLO* gene were detected, which suggests that this gene, or the genes in close proximity are involved in maternal calving difficulty. The *PCLO* gene is involved in human developmental malformations [37]. Other morphological traits, such as body depth, bone percentage, and birth weight have also been reported to be associated with loci in this region [38], which supports the important putative structural function of this region in cattle.

Furthermore, the meta-analysis results revealed that the association between *PDYN* on chromosome 13 and maternal calving difficulty was present in the three breeds, which is consistent with the intuitive perception that calving difficulty is one of the most painful conditions a cow can experience. Indeed, *PDYN* was shown to be linked to behaviour, pain perception, and psychological processes, and expression or up-regulation of this hormone serves as a biological indicator of stress in cattle [39, 40]. Detecting an association between a gene that encodes such a hormone and maternal calving difficulty confirms that this trait should remain a welfare concern.

### Perinatal mortality

The identification of several weak genomic associations with direct perinatal mortality suggests that the infinitesimal model hypothesis may indeed hold for this trait. Therefore, to achieve genetic improvement, we suggest analysing all loci collectively when assessing perinatal mortality since the trait is influenced by the cumulative effect of thousands of loci. The genes that were found to be associated with perinatal mortality in this study (*SLC20A1* and *UST*), were previously shown to be mainly involved with morphological abnormalities [41, 42], which suggests that structural deformities contribute to genetic variation in perinatal mortality. However, the proportion of genetic variance accounted for by loci in the vicinity of these genes was very small.

### Across-breed associations

We identified several breed-specific associations of moderate effect for calving performance due to either different breed allele substitution effects or to varying linkage phase between the genotyped allele and the causal mutation in each breed, which may have implications for across-breed genomic evaluations. The substantial difference in direction of allele effects between breeds may be the reason why across-breed genomic evaluations have been elusive until now [43]. In addition, such differences in allele effects suggest that genomic selection for such alleles could be deleterious for breeds that are either not represented, or poorly represented, in the reference populations if allele effects in that population and the reference population have opposite directions. This probably contributes to the negative correlations that are sometimes observed between direct genomic values for a breed which was not represented in the reference population [43]. Nevertheless, genomic regions that are common across breeds (with identical directions of allele effects) and that are significantly associated with calving difficulty were identified. These findings may help improve across-breed genomic evaluations. This suggests that a portion of the genomic variation that is attributed to calving performance is common to the three breeds. Partitioning genomic prediction algorithms into a within-breed component and an across-breed component may allow for these within-breed effects to be captured.

## Conclusion

Several strong associations for three calving performance traits in Holstein-Friesian, Charolais and Limousin populations were detected, although breed-specific SNP effects exist. These traits are polygenic traits since they are under the control of a large number of SNPs, each SNP being weakly associated with calving performance. Several previous studies have suggested the presence of a causal mutation on chromosome 18 for direct calving difficulty in dairy cattle. Although we did not detect any definitive mutation, a missense variant in the *CTU1* gene may contribute to the genetic variance of this trait. In addition, although the strongest associations for calving performance differed between breeds, genomic associations common to the three breeds were identified, which indicates that a portion of the genomic variation that is attributed to calving performance is common to the three breeds, which may have implications for across-breed genomic evaluations.

## Additional files

Additional file 1: Table S1. Bayes Factors (BF) and single-SNP regression p values for all SNPs with a BF greater than 200 for direct and maternal calving difficulty and a BF greater than 40 for perinatal mortality. Description: Table S1 provides details on the strongest associations identified for direct and maternal calving difficulty and direct perinatal mortality within each breed separately. The table includes breed, name, chromosome and position of each SNP, its minor allele frequency and the corresponding Bayes factor value from the Bayesian analyses and p-value from the single-SNP regression. **Table S2.** Gene symbols cited within the manuscript and their corresponding gene name. Description: A list of all genes mentioned throughout the manuscript and their corresponding gene name. Additional file 2: Figure S1. Single-SNP regression (SSR) results using imputed sequence data for direct calving difficulty in the Holstein-Friesian population. Description: Additional plots that show single-SNP regression results from the analysis of imputed sequence data on chromosomes 6, 10 and 2 for direct calving difficulty and chromosomes 4 for maternal calving difficulty.
