# Supplementary material for: Genome-wide association study for calving performance using high-density genotypes in dairy and beef cattle
Source: Genet Sel Evol. 2015 Jun 12;47(1):47. doi: 10.1186/s12711-015-0126-4 (PMC4464877; doi:10.1186/s12711-015-0126-4)
Supplement: Additional file 1: Table S1. — Bayes Factors (BF) and single-SNP regression p values for all SNPs with a BF greater than 200 for direct and maternal calving difficulty and a BF greater than 40 for perinatal mortality. Description: Table S1 provides details on the strongest associations identified for direct and maternal calving difficulty and direct perinatal mortality within each breed separately. The table includes breed, name, chromosome and position of each SNP, its minor allele frequency and the corresponding Bayes factor value from the Bayesian analyses and p-value from the single-SNP regression. Table S2. Gene symbols cited within the manuscript and their corresponding gene name. Description: A list of all genes mentioned throughout the manuscript and their corresponding gene name. [file 12711_2015_126_MOESM1_ESM.pdf]

Additional file 1

Table S1: Bayes Factors (BF) and single-SNP regression p values for all SNPs with a BF greater than 200 for direct and maternal calving difficulty and a BF greater than 40 for perinatal mortality

	SNP index	SNP name	Chr	Position	MAF	BF	P value
**Direct Calving Difficulty**							
Holstein-Friesian	4011	ARS-BFGL-NGS-109285	18	57589121	0.08	1684.26	8.95×10^{-10}
	525264	BovineHD1800016761	18	57548213	0.09	1523.56	8.95×10^{-10}
	741177	BovineHD4100008420	10	101721285	0.41	390.31	1.03×10^{-5}
	378664	BovineHD1200001026	12	3213665	0.25	253.84	3.37×10^{-4}
	168233	BovineHD0500000940	5	3768126	0.47	232.98	1.45×10^{-4}
	217768	BovineHD0600020010	6	72022013	0.38	223.73	9.91×10^{-8}
	168231	BovineHD0500000938	5	3764573	0.47	219.46	1.24×10^{-3}
Charolais	67815	BovineHD0200001668	2	5725051	0.13	3310.78	1.59×10^{-11}
	544037	BovineHD1900018321	19	63376818	0.47	1561.23	3.16×10^{-6}
	544036	BovineHD1900018320	19	63376120	0.47	907.81	3.16×10^{-6}
	533956	BovineHD1900007134	19	24561933	0.22	762.86	1.06×10^{-6}
	487931	BovineHD1600022505	16	77709903	0.39	709.33	1.38×10^{-5}
	761092	BTB-01124458	13	6683656	0.39	620.87	1.21×10^{-4}
	67816	BovineHD0200001669	2	5731378	0.13	383.88	1.46×10^{-11}
	398587	BovineHD1200024296	12	83902896	0.14	370.53	4.96×10^{-6}
	133777	BovineHD0300034147	3	117328152	0.39	295.66	5.01×10^{-4}
	253187	BovineHD0700024774	7	84451146	0.27	267.28	1.63×10^{-5}
	645696	BovineHD2600004234	26	16849292	0.36	234.46	1.69×10^{-6}
	631615	BovineHD2500001020	25	4516912	0.17	229.69	8.5×10^{-5}
	471782	BovineHD1600005037	16	18561778	0.37	213.47	5.57×10^{-5}
	645695	BovineHD2600004233	26	16848801	0.36	209.57	1.69×10^{-6}
Limousin	103101	BovineHD0300000138	3	657019	0.47	633.42	1.80×10^{-6}
**Maternal calving difficulty**							
Holstein-Friesian	544242	BovineHD1900018551	19	1942110	0.36	474.13	$8.63 \text{ X } 10^{-5}$
	416314	BovineHD1300016191	13	56557875	0.14	220.36	$4.18 \text{ X } 10^{-11}$
Charolais	126362	BovineHD0300026067	3	90597805	0.45	769.66	3.85×10^{-6}
	126363	BovineHD0300026068	3	90600368	0.47	700.57	4.85×10^{-6}
	126361	BovineHD0300026066	3	90596779	0.45	438.06	7.18×10^{-6}
	88704	BovineHD0200024474	2	85841490	0.27	306.54	9.47×10^{-7}
	88708	BovineHD0200024478	2	85872885	0.27	260.51	1.38×10^{-6}
	88688	BovineHD0200024458	2	85764305	0.27	241.09	9.47×10^{-7}
	252315	BovineHD0700023816	7	81801829	0.38	215.81	1.09×10^{-5}
	770652	Hapmap42056-BTA-108680	3	91136875	0.38	206.24	1.23×10^{-4}
**Direct Perinatal Mortality**							
Holstein-Friesian	644942	BovineHD2600003337	26	12797274	0.32	40.25	8.90×10^{-7}
Charolais	198553	BovineHD0500035019	5	119896486	0.36	52.13	2.83×10^{-6}
Limousin	750289	BovineHD4100018377	27	37032811	0.24	127.36	6.62×10^{-8}
	664893	BovineHD2700010585	27	37023628	0.23	92.22	1.33×10^{-7}
	664891	BovineHD2700010583	27	37021710	0.23	88.02	1.33×10^{-7}
	664898	BovineHD2700010590	27	37031485	0.23	87.06	1.33×10^{-7}
	664897	BovineHD2700010589	27	37030084	0.23	84.49	1.33×10^{-7}
	664890	BovineHD2700010582	27	37019464	0.23	78.42	1.33×10^{-6}
	664895	BovineHD2700010587	27	37027959	0.23	77.78	1.33×10^{-7}
	664894	BovineHD2700010586	27	37026496	0.24	75.56	1.33×10^{-7}

	664899	BovineHD2700010591	27	37034231	0.23	72.55	1.33×10^{-7}
	664885	BovineHD2700010577	27	37007637	0.24	69.40	7.69×10^{-8}
	664888	BovineHD2700010580	27	37015031	0.24	67.05	7.69×10^{-8}

Table S2:Gene symbols cited within the manuscript and their corresponding gene name

**Gene Symbol**	**Corresponding Name**
ANKAR	Ankyrin and armadillo repeat containing
ASNSD1	Asparagine synthetase domain containing 1
C2H2orf88	Chromosome 2 open reading frame, human C2orf88
CEACAM18	Carcinoembryonic antigen-related cell adhesion molecule 18
CLOCK	Clock circadian regulator
CORIN	Corin, serine peptidase
CROT	Carnitine O-octanoyltransferase
CTU1	Cytosolic thiouridylase subunit 1
DCAF6	DDB1 and CUL4 associated factor 6
DENND6B	DENN/MADD domain containing 6B
ENSBTAG00000004082	Novel gene
ENSBTAG00000019276	Novel gene
HDAC10	Histone deacetylase 10
HGF	Hepatocyte growth factor (hepapoietin A; scatter factor)
HIBCH	3-hydroxyisobutyryl-CoA hydrolase
INPP1	Inositol polyphosphate-1-phosphatase
KIAA1324-like	KIAA1324-likeortholog
MAPK11	Mitogen-activated protein kinase 11
MAPK12	Mitogen-activated protein kinase 12
MFSD6	Major facilitator superfamily domain containing 6
MSTN	Myostatin
ORMDL1	ORMDL sphingolipid biosynthesis regulator 1
OSGEPL1	O-sialoglycoproteinendopeptidase-like 1, mRNA
PCLO	Piccolo presynaptic cytomatrix protein
PDYN	Prodynorphin
PLXNB2	Plexin B2
PMS1	PMS1postmeiotic segregation increased 1
PPP6R2	Protein phosphatase 6, regulatory subunit 2
PRKCA	Protein kinase C, alpha
SEMA3D	Sema domain, immunoglobulin domain (Ig), short basic domain, secreted, (semaphorin) 3D
SIGLEC12	Sialic acid binding Ig-like lectin 12
SIRPA	Signal-regulatory protein alpha
SLC20A1	Solute carrier family 20 (phosphate transporter), member 1
TMEM194B	Transmembrane protein 194B
UST	Uronyl-2-sulfotransferase
ZNF615	Zinc finger protein 615
