# Supplementary material for: Genome-wide association study for calving performance using high-density genotypes in dairy and beef cattle
Source: Genet Sel Evol. 2015 Jun 12;47(1):47. doi: 10.1186/s12711-015-0126-4 (PMC4464877; doi:10.1186/s12711-015-0126-4)
Supplement: Additional file 2: Figure S1. — Single-SNP regression (SSR) results using imputed sequence data for direct calving difficulty in the Holstein-Friesian population. Description: Additional plots that show single-SNP regression results from the analysis of imputed sequence data on chromosomes 6, 10 and 2 for direct calving difficulty and chromosomes 4 for maternal calving difficulty. [file 12711_2015_126_MOESM2_ESM.pdf]

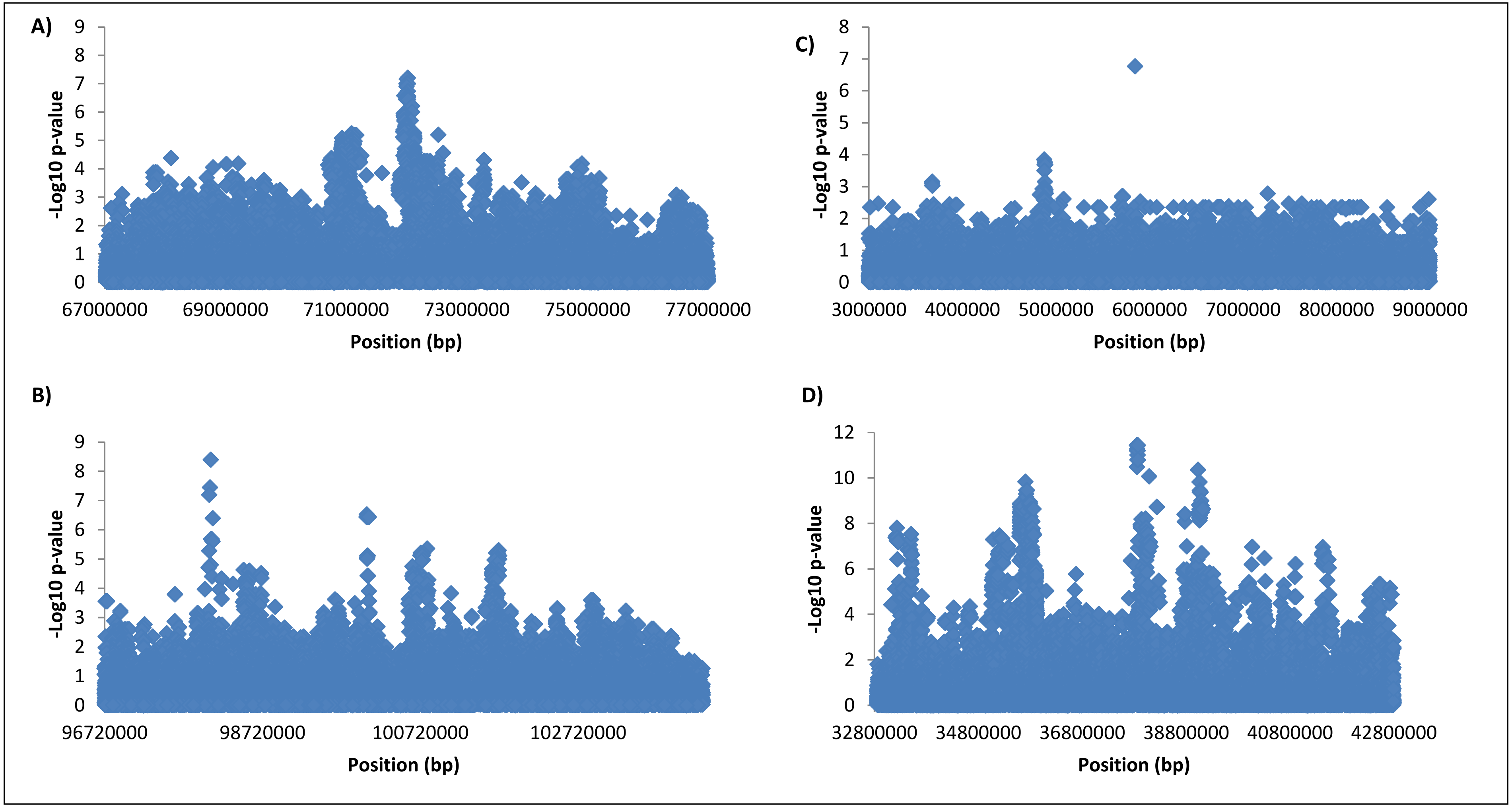

Additional file 2. Single SNP regression (SSR) results using imputed sequence data for direct calving difficulty in the Holstein-Friesian population. A) Chromosome 6 (10Mb region surrounding HD peak at 72Mb) B) chromosome 10 (8 Mb region surrounding HD peak at 101Mb) C) chromosome 2 (6Mb region surrounding the HD peak at 5.6 to 6.6Mb found in the Charolais and Limousin population) and D) SSR results for maternal calving difficulty on chromosome 4 (10Mb region surrounding peak at 37Mb).
